# CD95 maintains stem cell-like and non-classical EMT programs in primary human glioblastoma cells

**DOI:** 10.1038/cddis.2016.102

**Published:** 2016-04-28

**Authors:** M Drachsler, S Kleber, A Mateos, K Volk, N Mohr, S Chen, B Cirovic, J Tüttenberg, C Gieffers, J Sykora, C R Wirtz, W Mueller, M Synowitz, A Martin-Villalba

**Affiliations:** 1 Department of Molecular Neurobiology, German Cancer Research Center (DKFZ), Heidelberg 69120, Germany; 2 Department of Cell Differentiation and Tumorigenesis, Max Delbrück Center, Berlin 13125, Germany; 3 Department of Neurosurgery, Clinical Center Idar-Oberstein, Idar-Oberstein 55743, Germany; 4 Apogenix GmbH, Heidelberg 69120, Germany; 5 Department of Neurosurgery, University Hospital Ulm, Günzburg 89312, Germany; 6 Department of Neuropathology, University Hospital Leipzig, Leipzig 04103, Germany; 7 Department of Neurosurgery, Charité, University Medicine Berlin, Berlin 13353, Germany; 8 Department of Neurosurgery, Campus Kiel, Kiel 24105, Germany

## Abstract

Glioblastoma (GBM) is one of the most aggressive types of cancer with limited therapeutic options and unfavorable prognosis. Stemness and non-classical epithelial-to-mesenchymal transition (ncEMT) features underlie the switch from normal to neoplastic states as well as resistance of tumor clones to current therapies. Therefore, identification of ligand/receptor systems maintaining this privileged state is needed to devise efficient cancer therapies. In this study, we show that the expression of CD95 associates with stemness and EMT features in GBM tumors and cells and serves as a prognostic biomarker. CD95 expression increases in tumors and with tumor relapse as compared with non-tumor tissue. Recruitment of the activating PI3K subunit, p85, to CD95 death domain is required for maintenance of EMT-related transcripts. A combination of the current GBM therapy, temozolomide, with a CD95 inhibitor dramatically abrogates tumor sphere formation. This study molecularly dissects the role of CD95 in GBM cells and contributes the rational for CD95 inhibition as a GBM therapy.

Recent studies have identified a highly tumorigenic population of cancer cells with stem cell-like properties, often termed cancer stem cells (CSCs), in mouse models of a variety of solid tumors.^1, 2, 3^ These studies define CSCs as a restricted population of cells with extensive clonogenic potential that generate more ‘differentiated' progeny with reduced long-term proliferative capacity. The acquisition and maintenance of a stem cell-like state by cancer cells has been linked to the process of epithelial-to-mesenchymal transition (EMT).^4, 5^ Because of their intrinsic resistance to radiotherapy and chemotherapy, CSCs can replenish a tumor after an initially successful therapy.^1, 6^ Thus CSCs and their microenvironment appear as attractive therapeutic targets to eliminate the repository potential of a tumor.

In order to design CSC-based therapies in the clinical setting, reliable surface markers for the identification of CSCs need to be established. In case of glioblastoma (GBM), a plethora of such markers, including Prominin (CD133), stage-specific embryonic antigen 1 (CD15), Integrin α6 (ITGA6), CD44, Ephrin A2 (EphA2), Ephrin A3 (EphA3) and myeloid elf-1-derived factor (MEF), has been proposed.^7, 8, 9, 10, 11, 12, 13^ However, surface marker-negative GBM cells are also able to effectively initiate tumor growth, and therefore, great caution is recommended when designating a marker-positive cell as a GBM stem cell (GSC).^11, 14, 15^

CD95 (also known as FAS or APO-1) came to the fore in 1989 as a potential therapeutic target in cancer owing to its function as a trigger of apoptosis.^16, 17^ Activation of CD95 leads to recruitment and activation of caspases that irreversibly induce apoptosis.^18^ In addition, phosphorylation of tyrosine within CD95 intracellular death domain has been observed following binding by CD95 ligand (CD95L).^19, 20^

Extensive characterization of the role of CD95 in cancer has, however, revealed that malignant tumor cells are generally resistant to CD95-induced apoptosis. Instead, activation of CD95 in a variety of solid tumors increases motility and invasion of tumor cells.^19^ In GBM, invasive migration of tumor cells is mediated by downstream signaling via Yes and PI3K and can be significantly reduced by inhibition of CD95 activation.^20^ Indeed, CD95 is required for optimal cancer cell growth and migration while inhibition of CD95 signaling in established epithelial tumors induces cancer cell death.^21, 22, 23^ In breast cancer, CD95/CD95L signaling promotes proliferation of a population of CSCs.^24^ Non-apoptotic CD95 signaling is also observed under non-malignant conditions. In neural stem cells (NSCs), activation of CD95 increases survival and activation for injury-induced brain repair.^25^

Considering these observations, we sought to elucidate whether CD95 signaling might also activate or maintain a stem cell-like and EMT-programmed population of cells in GBM.

## Results

### CD95 is overexpressed and can serve as a prognostic biomarker in GBM

Molecular markers have been identified in almost every type of cancer and can aid in the estimation of a patient's response to treatment and prognosis.

**Figure 1:**
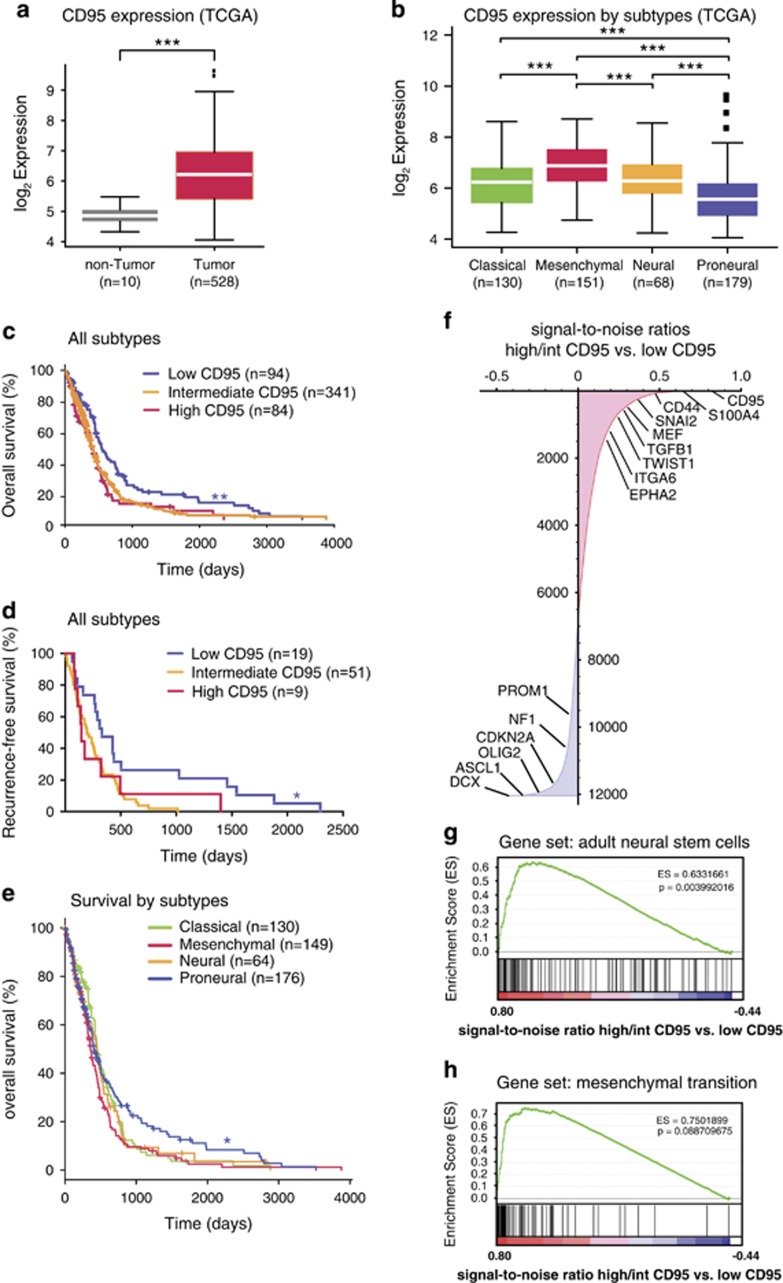
CD95 is a prognostic biomarker in GBM patients and linked to stem cell and EMT gene expression patterns. (**a**) CD95 expression in TCGA GBM samples compared with unmatched non-tumor control tissue (Wilcoxon test). (**b**) CD95 expression compared between distinct GBM subtypes in the TCGA data set (Wilcoxon test with Holm correction). (**c**) Overall survival in TCGA GBM patients stratified according to CD95 expression. (**d**) Progression-free survival in TCGA GBM patients stratified according to CD95 expression (log-rank test). (**e**) Overall survival in TCGA GBM patients grouped by subtype (log-rank test). (**f**) TCGA data set genes ranked according to signal-to-noise ratios for CD95^high^/CD95^int^ *versus* CD95^low^ expression groups. (positive values (red): higher expression in the CD95^high/int^ group; negative values (blue): higher expression in the CD95^low^ group). (**g**) GSEA for enrichment of an adult NSC gene signature in the ranked TCGA gene data set. (**h**) GSEA for enrichment of an EMT gene signature in the ranked TCGA gene data set

To get insight into the role of CD95 in GBM, we analyzed a data set accessible via The Cancer Genome Atlas (TCGA) featuring expression as well as clinical patient data.^26^ When compared with unmatched, non-tumor controls, CD95 was found to be highly overexpressed in GBM patient samples (Figure 1a). Based on their respective genomic and RNA signatures, four distinct subtypes (classical, mesenchymal, neural and proneural) have been proposed for GBM.^27^ When classified according to these subtypes, CD95 was predominantly expressed in mesenchymal tumors in the TCGA GBM data set while CD95 expression was the lowest in proneural GBM (Figure 1b).

Correlation of tumor's CD95 expression with clinical data showed that CD95 might function as a prognostic molecular marker in GBM patients. Low tumor CD95 expression was associated with significantly longer overall survival (Figure 1c). When our analysis was limited to patients in whom complete remission of the tumor was achieved by initial therapy, similar observations were made. Interestingly, this feature seems to be shared by other TNF receptor family members, as the expression levels of TNFRSF1A,B; TNFRSF11B; TNFRSF12A and TNFRSF14 showed a similar trend toward longer overall survival in the patients with low TNFR expression (Supplementary Figure S1). Again, low tumor CD95 expression was associated with a significantly increased time to tumor recurrence after initial therapy (Figure 1d). Of note, almost all tumors classified as low CD95 expression GBM belonged to the proneural subtype. Consequently, significantly longer overall survival was also observed for patients with proneural tumors relative to other GBM subtypes (Figure 1e and Supplementary Figure S2).

### Expression of CD95 correlates with NSC and EMT gene expression signatures

To gain deeper insight into the processes driving the aggressive phenotype associated with the expression of CD95, we performed gene set enrichment analysis (GSEA).^28^ The genes in the TCGA data set were ranked according to their association with high and intermediate *versus* low CD95-expressing GBM.

At first glance, known GSC markers, including MEF, EPHA2, ITGA6 and CD44 as well as major EMT regulators appeared to be highly overexpressed in tumors with high or intermediate CD95 expression (Figure 1f and Supplementary Table S1). In contrast, DCX, ASCL1 and OLIG2, genes normally expressed by more differentiated neuronal progeny as well as cell cycle regulators CDKN2A and NF1, were preferentially expressed in the less aggressive tumors expressing low levels of CD95 (Figure 1f). GSEA showed a significant enrichment of a NSC gene signature^29^ in high and intermediate CD95-expressing tumors (Figure 1g). Similarly, a published EMT gene signature^30^ was also enriched in this group of highly aggressive GBM (Figure 1h).

### CD95^high^ GBM cells display CSC properties *in vitro*

**Figure 2:**
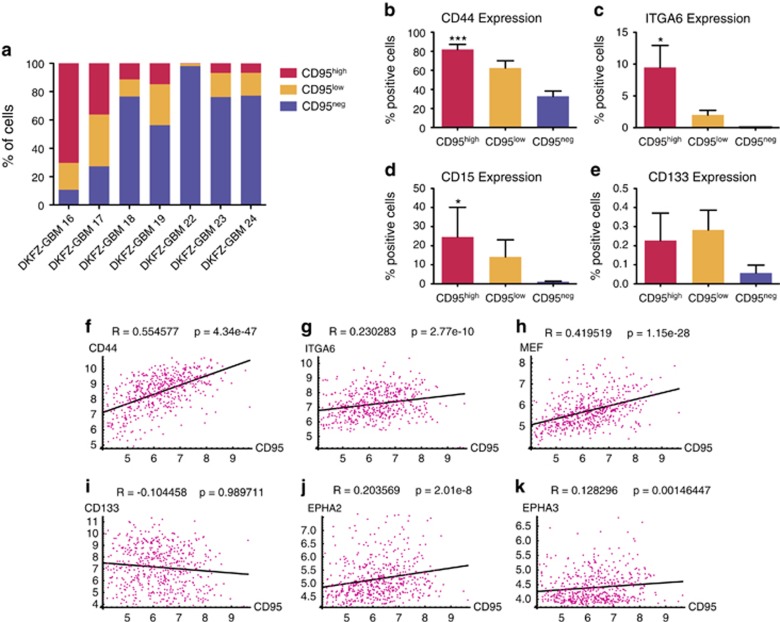
CD95^high^ GBM cells co-express known GSC markers. (**a**) CD95 expression in seven patient tumor samples analyzed by flow cytometry. (**b**–**e**) Co-expression of known GSC markers CD44 (*n*=7), Integrin α6 (ITGA6; *n*=7), CD15 (*n*=3) and CD133 (*n*=7) within the patient samples (Friedman test). (**f**–**k**) Correlation of CD95 expression and known GCS markers in the TCGA GBM data set. (*n*=519 patients, *R*: Pearson's correlation coefficient, *P*: probability for no or negative correlation)

To further investigate the properties of CD95-expressing GBM cells *in vitro*, tumor cells were freshly isolated from seven surgical specimens and analyzed by flow cytometry (FACS). A population of cells with high levels of CD95 surface expression (CD95^high^) – ranging from 0.2 to 71% – was found in each sample (Figure 2a). Co-staining for known GSC surface markers revealed a significant enrichment of CD44, ITGA6 and CD15 but not CD133-expressing cells within this CD95^high^ GBM cell population (Figures 2b–e). To test this enrichment of known GSC markers in a larger patient cohort, we again turned to the TCGA GBM patient data set. Paralleling our findings in primary GBM cells, a significant correlation was observed between the expression of CD95 and the known GSC markers CD44, ITGA6, MEF, EPHA2 and EPHA3 but not for CD133 in this data set (Figures 2f–k). Because of the large number of patients (*n*=519), all correlations were statistically significant. Nevertheless, correlation was strongest between the expression of CD95 and CD44 as well as MEF (Figures 2f and h).

**Figure 3:**
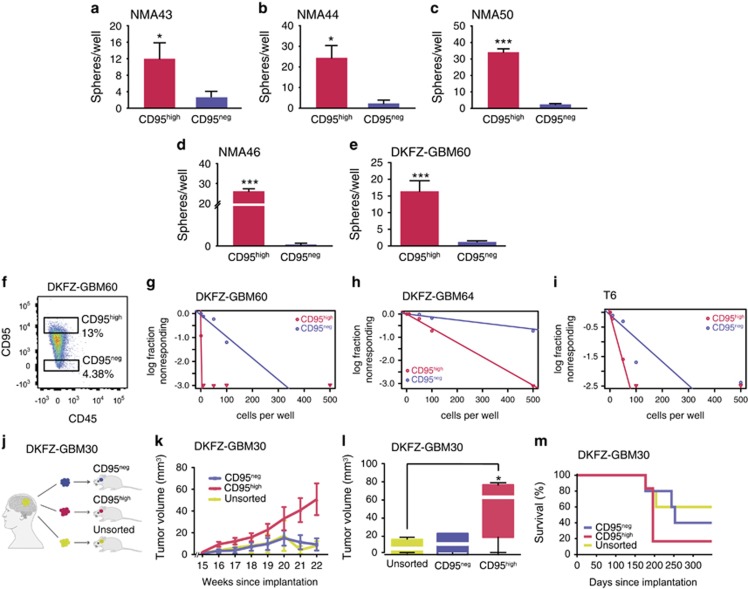
CD95^high^ GBM cells display superior self-renewal *in vitro* and are highly tumorigenic *in vivo*. (**a**–**e**) Sphere formation by FACS-sorted CD95^high^ and CD95^neg^ cells freshly isolated from five patient tumor samples plated at 500 cells per well (*t*-test). (**f**) Representative gating strategy for cell sorting. (**g**–**i**) Sphere formation by CD95^high^ and CD95^neg^ cells freshly isolated from three tumor samples plated under limiting-dilution conditions. Estimated stem cell frequencies: (L) CD95^neg^: 1 : 110, CD95^high^: 1 : 1 (limiting dilution not reached); (M) CD95^neg^: 1 : 771, CD95^high^: 1 : 163; (N) CD95^neg^: 1 : 122, CD95^high^: 1 : 30. (**j**) 1 × 10^3^ unsorted, CD95^neg^ or CD95^high^ cells were injected into the striatum of Fox Chase SCID beige mice (*n*=5 for unsorted and CD95^neg^, *n*=6 for CD95^high^). (**k**) Tumor growth was monitored by T2-weighted MRI. (**l**) Tumor volumes at the end of the MRI observation period 22 weeks after injection (ANOVA). (**m**) Kaplan–Meier curve showing the survival of the animals after the MRI observation period. In accordance with the observed tumor growth, CD95^high^-injected animals show the highest mortality

These observations suggested that the CD95^high^ GBM cell population might possibly hold certain stem cell-like traits. We therefore tested the self-renewal capacity of FACS-sorted cells isolated from five patient samples by measuring sphere formation. CD95^high^ cells showed extensive sphere-formation capacities, while sphere formation in CD95^neg^ cells was very limited (Figures 3a–e). The same was also true when the cells were plated under limiting-dilution conditions (Figures 3f–i). Even under highly restrictive conditions down to one cell per well, CD95^high^ GBM cells showed profound sphere-formation capacities. The estimated stem cell frequencies ranged from 1 : 1 to 1 : 163 for CD95^high^ compared with 1 : 110 to 1 : 773 for CD95^neg^ GBM cells (Figures 3g–i).

### CD95^high^ GBM cells are highly tumorigenic *in vivo*

In order to further investigate these observed properties of CD95^high^ cells *in vivo*, primary cells were isolated from a human sample, depleted of CD45-positive immune cells as well as CD31-positive endothelial cells and sorted for CD95 expression. One thousand unsorted, CD95^high^ or CD95^neg^ primary cells were injected into the striatum of SCID beige mice (Figure 3j). The CD95^high^ fraction amounted to 3.33% while CD95^neg^ cells made up 52.4% of cells in the patient sample (Supplementary Figure S3). Tumor incidence was 86 and 55% for CD95^high^ and CD95^neg^ xenografts, respectively. Sixteen weeks after xenotransplantation, first lesions were detected in T2-weighted magnetic resonance imaging (MRI) sequences (Figure 3k). From then on, the CD95^high^-expressing tumors showed sustained growth throughout the observation period of a further 7 weeks (Figure 3k). In contrast, despite being able to initiate a tumor, CD95^neg^ and unsorted tumor cells were not able to sustain tumor growth. After an initial growth phase of the detectable lesions, the size of the lesion stagnated or even decreased (Figure 3k). At the end of the MRI observation period 22 weeks after implantation, CD95^high^ GBM cells had given rise to significantly larger tumors than CD95^neg^ or unsorted cells (Figure 3l and Supplementary Movies SM1 and SM2). The MRI-measured tumor volumes were confirmed by histopathological analysis in samples of tumor-bearing brains (Supplementary Figure S3). Paralleling the significantly larger tumor in the CD95^high^-injected group, those animals also showed a higher mortality (Figure 3m).

To confirm these results, orthotopic xenotransplantation was repeated with cells derived from a second, independent patient sample. Because of the limited availability of primary patient material, tumor cells were expanded by two rounds of subcutaneous xenografting (Supplementary Figure S3). Despite the increasingly efficient tumor formation by CD95^neg^ cells, CD95^high^ cells once more sustained superior tumor growth (Supplementary Figure S3).

### Inhibition of CD95/CD95L signaling precludes sphere formation while CD95 and CD95L are upregulated by conventional therapy

**Figure 4:**
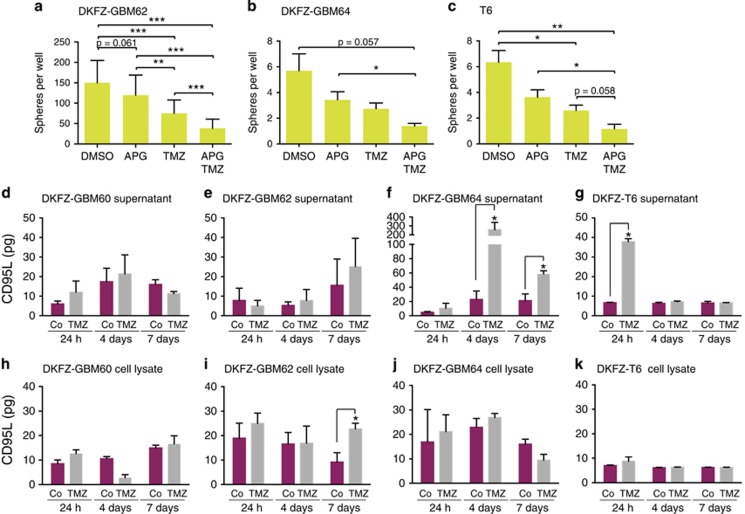
Sphere formation is dependent on CD95/CD95L signaling. (**a**–**c**) Sphere formation by freshly isolated cells from three patient tumor samples treated with DMSO (control), APG101 (APG), TMZ or a combination of both (*n*≥12 per conditions, overall comparison: one-way ANOVA, pairwise comparison: Tukey's multiple comparison test). (**d**–**k**) CD95L-ELISA for patient-derived cell culture supernatants (**d**–**g**) or lysates (**h**–**k**) treated with DMSO (Co) or TMZ (two sided *t*-test with Holm correction)

Next we asked whether self-renewal of GBM cells might be dependent on a CD95/CD95L signal. We therefore treated freshly isolated GBM cells with APG101 (Apocept), a CD95-Fc fusion protein designed to scavenge CD95L and thereby block CD95 activity. In addition, cells were treated with the GBM standard chemotherapy, temozolomide (TMZ) or a combination of APG101 and TMZ. APG101 was able to significantly reduce GBM sphere formation in combination with TMZ but not alone (Figures 4a–c). As the inhibitor effect of APG101 is based on its binding to CD95L, we reasoned that expression of CD95L by GBM cells under culture conditions might be low and increased by TMZ treatment. To test this hypothesis, we measured CD95L by ELISA in the supernatant and lysates of GBM cells treated with TMZ or left untreated. In two out of the four samples tested, TMZ treatment resulted in an increase in CD95L expression while a similar trend was observed in the remaining samples. This could explain the inhibitory self-renewal effect by the combination of TMZ and APG101 (Figures 4d–k).

**Figure 5:**
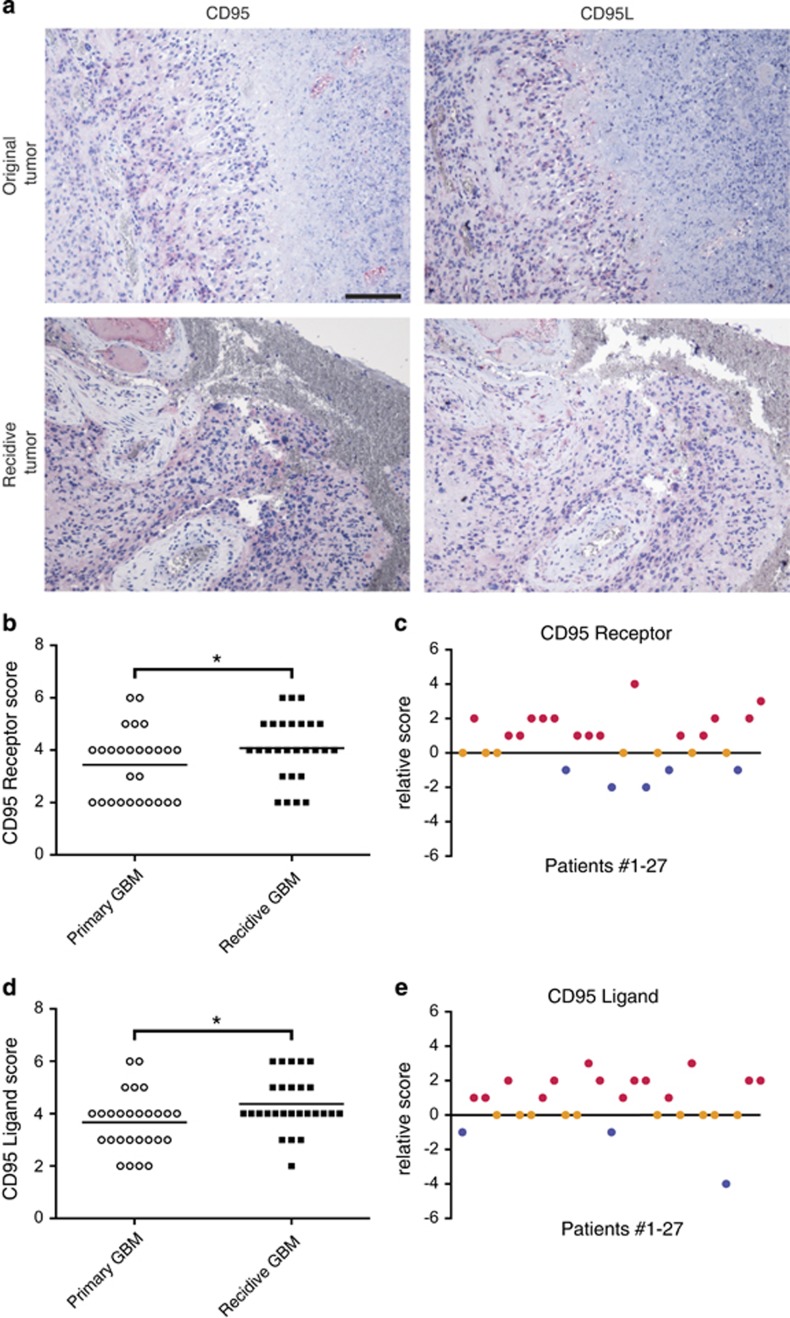
Expression of CD95 and CD95L is increasing in recurrent GBM. (**a**) Anti-CD95 and -CD95L immunohistochemistry (IHC) of primary and matching relapsing tumors (representative images, scale bar: 200 *μ*m). (**b**) Scoring results for anti-CD95 IHC of 27 primary and relapsing tumors. (Wilcoxon test). (**c**) Individual relative CD95 scores (relapse score – primary tumor score) for the 27 patients showing an overall increase of CD95 expression in the relapsing tumors (average relative score=0.70, 95% Cl: 0.14–1.27). (**d**) Scoring results for anti-CD95L IHC of 27 primary and relapsing tumors. (Wilcoxon test). (**e**) Individual relative CD95L scores for 27 patients showing an overall increase in CD95L expression in the relapsing tumors (average relative score=0.73, 95% CI: 0.13–1.33)

To compare these *in vitro* findings to the clinical setting, protein expression of CD95 and CD95L was examined in tissue sections from 27 original and matched relapse tumors (Figure 5a and Supplementary Table S2). For quantification purposes, the different tumors were scored on a scale ranging from two (lowest) to six (highest) according to the intensity and area of CD95 and CD95L immunohistochemical staining across the different tumor sections. After initial treatment, CD95 expression was significantly upregulated in relapse tumors (Figures 5b and c). These results point toward the occurrence of a selection process favoring CD95-expressing cells with increased chemoresistance. In accordance with our findings *in vitro*, expression of CD95L was also upregulated in relapsing GBM after initial treatment of the original tumor (Figures 5d and e).

### Tyrosine phosphorylation of CD95 recruits P85 and SFK and is essential for maintenance of an EMT program in GBM cells

**Figure 6:**
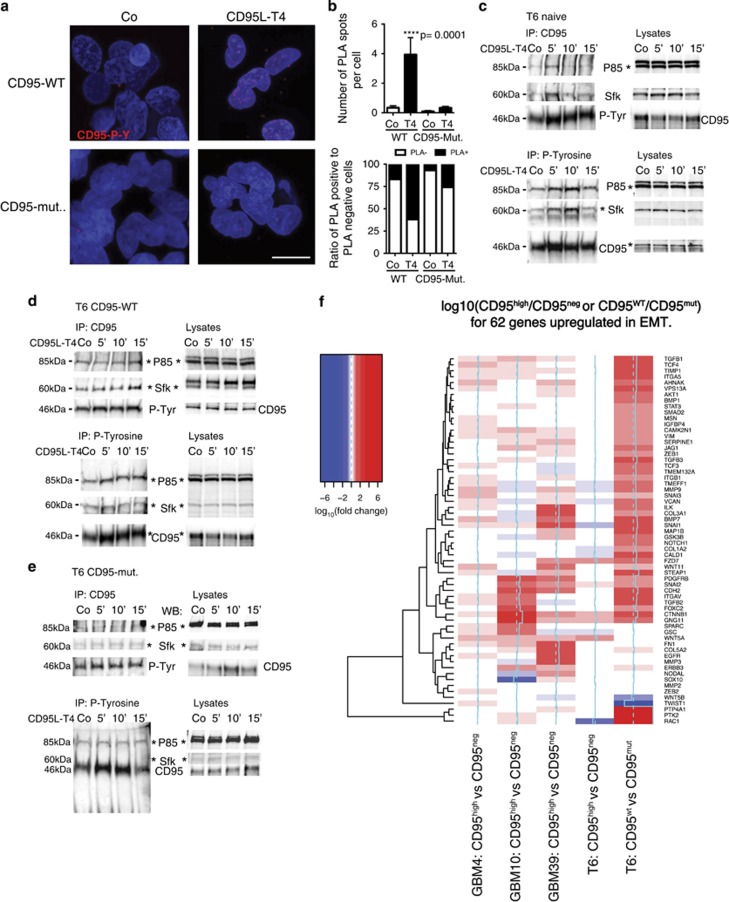
Tyrosine phosphorylation of CD95 recruits P85 and SFK and induces ncEMT. (**a**) PLA for tyrosine phosphorylation of the CD95 receptor in patient-derived CD95^neg^ GBM cells transduced with a CD95 wild-type (CD95-WT) or CD95 mutant (tyrosine-to-alanine, CD95-mut) lentivirus stimulated with PBS (Co) or CD95L-T4. (**b** and **c**) Quantification of PLA results (*t*-test). (**d** and **e**) IP for CD95 and P-Tyrosine GSCs in naive, CD95-WT and CD95-mut GBM cells stimulated with CD95L-T4. Blots were probed with anti-P85 (regulatory PI3K subunit), anti-Sfk, anti-CD95 or anti-P-Tyrosine antibodies, respectively. (**f**) Expression of 62 genes expected to be upregulated during ncEMT measured by qPCR array in four FACS sorted as well as in a lentivirally transduced patient samples. Heat map displays log10 (CD95^high^ or CD95^wt^)−log10 (CD95^neg^ or CD95^mut^) values

We have previously suggested that phosphorylation of a tyrosine residue lying within CD95's intracellular death domain underlies non-apoptotic downstream signals. This hypothesis was, however, based on *in silico* prediction but never demonstrated at the molecular level. To study tyrosine phosphorylation in GBM, we transduced CD95-negative (CD95^neg^) cells with lentiviral vectors entailing either a CD95 wild type (CD95-WT) or a CD95 tyrosine-to-alanine mutant (CD95-mut) that precludes phosphorylation. To detect tyrosine phosphorylation of CD95 *in situ*, we used a proximity ligation assay (PLA) that uses antibody–DNA conjugates to enable the conversion of protein–protein interactions (<40 nm distance) into detectable DNA molecules. Thus a phosphorylated tyrosine in CD95 can be detected by the proximity of anti-CD95 to anti-phospho-tyrosine antibodies in GBM cells. Indeed, this assay revealed the presence of tyrosine phosphorylation of CD95 (Figure 6a). Upon stimulation with CD95L-T4, CD95 tyrosine phosphorylation strongly increased in CD95-WT but not in CD95-mut cells (Figures 6a and b). After establishing this cellular model, we next wanted to test the recruitment of known downstream mediators of non-apoptotic CD95 signals. Association of the regulatory PI3K subunit, p85, as well as a Src-familiy-kinase member (Sfk) with CD95 was readily detected by CD95 and phospho-tyrosine immunoprecipitation (IP) in naive, unsorted GBM cells treated with CD95L (Figure 6c and Supplementary Figure S4). Upon stimulation in lentivirally transduced GBM cells, recruitment of P85 and Sfk was observed in CD95-WT but not in CD95-mut cells (Figures 6d and e). We thus conclude that, upon stimulation, tyrosine phosphorylation within the intracellular death domain might contribute to the assembly of the non-apoptotic CD95 signalosome in GBM cells.

We hypothesized that CD95 signaling, via tyrosine phosphorylation and recruitment of downstream mediators, might induce an EMT signal that can promote stemness and invasive migration in GBM cells. Fitting this hypothesis, expression of CD95 correlated with the expression of the known EMT marker genes VIM, FN1, SNAI2 and S100A4 in the TCGA data set (Supplementary Figure S3). To further investigate this potential link, we measured the expression of 62 EMT-associated genes in qPCR arrays for CD95^high^ *versus* CD95^neg^ GBM cells sorted from 4 individual patient samples as well as in our CD95-WT *versus* CD95-mut cells. Here EMT-associated genes were again upregulated in CD95^high^ cells (Figure 6f and Supplementary Table S4). Furthermore, the EMT-associated genes were strongly downregulated in CD95-mut cells lacking tyrosine phosphorylation within the death domain of CD95 (Figure 6f).

## Discussion

### An aggressive population of GBM cells with stem cell-like properties expresses high levels of CD95

Our results demonstrate that CD95 can be used for isolation of a highly aggressive population of GBM cells with certain stem cell-like features. CD95^high^ GBM cells exhibit superior self-renewal capacity *in vitro* and are enriched in relapsing tumors after conventional therapy. Blocking of CD95 signaling inhibits GBM cell self-renewal. In accordance with findings in adult NSCs as well as breast CSCs, CD95 thus seems to mediate an activation signal in CD95^high^ GBM cells too.^24, 25^

Examination of the TCGA data set revealed that CD95 was predominantly expressed in mesenchymal GBM subtype. Studies employing genetically engineered mouse models have shown that occurrence of genetic alterations in a certain cell type induces formation of distinct GBM subtype tumors.^31, 32^

In this study, the mesenchymal subtype arose from introducing mutations in differentiated neural cells. Thus an ongoing dedifferentiation process that confers a NSC- or progenitor cell-like phenotype was speculated as underlying tumor-initiation mechanism.^31^ Our results indicate that CD95 might be actively involved in this process by functioning as an EMT signal. In addition, inflammatory infiltrates are highly prevalent within mesenchymal GBM.^27^ As reported previously, innate immune cells rely on the CD95/CD95L system to be recruited to the central nervous system after injury.^33^ And lately, innate immunity has been linked to efficient reprogramming in the induction of pluripotency.^34^ It is thus possible that a signal mediated by CD95L-bearing immune cells can contribute to the establishment of an aggressive tumor phenotype. Additionally, we showed that expression of CD95L increases during tumor progression and after chemotherapy. This suggests that GBM cells themselves might be able to maintain CD95 activity as a stimulating signal, especially later on during disease progression.

### CD95, stemness and the EMT program

As a cell of tumor origin does not necessarily have to be a CSC, it is reasonable to assume that there must be a way for cancer cells to acquire stem cell traits.^35^ To this end, the developmental program of EMT has been reported to confer pro-invasive as well as stem cell-like properties to cancer cells.^4, 36^ Similarly, the EMT-mediating transcription factor Twist1 has been shown to be crucial to melanoma initiation and progression.^37^

Our observations indicate an ongoing process similar to EMT that maintains a population of aggressive, stem cell-like cells in GBM. Given the fact that this process is similar but not identical to the EMT process resulting in metastasis formation in carcinomas, it is appropriate to denote this process as non-classical EMT (ncEMT). CD95, via tyrosine phosphorylation of its intracellular death domain and subsequent downstream via the PI3K pathway, seems to be actively involved in this process. In line with these observations, the PI3K signaling pathway is involved in the induction of an EMT state in other tumors as well.^38^ CD95 belongs to the tumor necrosis factor alpha (TNF*α*) superfamily, and similarly, TNF*α* has been implicated in EMT, tumorigenesis and tumor cell invasion in various ways.^39^ Furthermore, inhibition of the major EMT-regulator TGF-*β* has been shown to inhibit CSC activity in GBM.^7^ Cells at the invasive front of GBM as well as other solid tumors usually acquire mesenchymal traits to promote their invasive migration.^20, 40, 41^ Another interesting aspect regarding the role of EMT in cancer has been highlighted recently. Studies in pancreatic and lung cancer have indicated that EMT might not be the main driver of metastatic growth but might rather induce a chemoresistant state in tumor cells.^42, 43^

Taken together, our findings suggest an active role of CD95 in GBM growth and progression. Beyond a function as a sheer surface marker, non-apoptotic signals mediated via phosphorylation within the intracellular death domain seem to regulate ncEMT in a manner similar to other TNF*α* family and tyrosine kinase receptors. However, additional efforts are required to further elucidate on the exact signaling pathway and its regulation.

### Implications for GBM therapy

Our results imply possible clinical applications of CD95 as a prognostic biomarker as well as a therapeutic target in GBM therapy. We identified low CD95 expression as a positive prognostic biomarker predicting favorable overall and recurrence-free survival.

In 2008, we discovered that CD95 induces invasive migration of GBM.^20^ Based on this findings, APG101 (Apocept), a CD95-Fc fusion protein that neutralizes CD95L, has been developed. In a first safety trial, APG101 showed no toxicity.^44^ Recently, the combination of APG101 and radiotherapy *versus* radiotherapy only in patients with relapsing GBM was tested in a Phase II clinical trial.^45^ Surpassing all expectations, application of the compound in combination with radiotherapy almost doubled the progression-free survival in these patients for whom only very limited therapeutic options are available.^45^ Increased overall survival was also observed upon APG101 treatment within a group of patients featuring low levels of DNA methylation within the CD95L promoter (and therefore expressing high levels of CD95L). Consequently, CD95L promoter methylation status was suggested as a biomarker to predict a tumor's response to APG101 treatment.^45^

Our current study provides additional insight into possible cellular effects of APG101. Given its supposed involvement in the regulation of ncEMT in GBM cells, CD95 might be able to specifically target the aggressive subpopulation of tumor cells that we have described here. Therefore, APG101 might be a valuable addition to conventional therapies targeting the tumor bulk and we are eager to see its effect in the larger-scale clinical trials to come.

## Materials and Methods

### TCGA data analysis

TCGA data was downloaded from the TCGA Data Portal (https://tcga-data.nci.nih.gov/), and level-3 data from the Affymetrix U133a microarray platform (Santa Clara, CA, USA) was used for gene expression analysis.

### Subtype annotation in the TCGA data set

Samples were annotated to the four subtypes (neural, proneural, mesenchymal and classical) defined by Verhaak *et al.*,^27^ using a supervised approach based on support vector machines (SVM).

A SVM model was trained with the 171 annotated samples and the 840 genes from the signatures published by Verhaak *et al.*^27^ using the svm() function from the R (http://cran.r-project.org) package e1071, with a radial kernel and default parameters.^27^ The predictive capacity of the adjusted model was estimated by 10-fold cross-validation on the training set, reaching a 91.23% total accuracy. The trained model was then used to predict the subtype of the 376 non-annotated tumor samples in the TCGA data set. These new annotated samples were added to the initial 171, adding up to 547 samples belonging to 528 patients. When there were multiple samples for a patient, the prediction was always consistent.

### Gene set enrichment analysis

GSEA was performed as described by Subramanian *et al.*^28^ NSC and ncEMT signatures were derived from the published data.^29, 30^

### Handling of patient tumors and tumorsphere cultures

Collection of patient samples was approved by the Ethics committee of the Charité University Medicine, Campus Virchow-Klinikum, Berlin (EA3/023/06) and the Medical Ethics committee II of the medical faculty Mannheim of the University of Heidelberg (EA2/101/08). All specimens were examined by a neuropathologist to confirm that each tumor met the WHO criteria for GBM. Samples were cut in small pieces, digested using Dispase, DNAse and Papain and cultured in neurobasal medium (see Supplementary Information) in an incubator at 37 °C and 5% CO_2_.

### Complete neurobasal medium for tumorsphere cultures

Neurobasal medium (Invitrogen, Thermo Fisher Scientific GmbH, Schwerte, Germany) supplemented with B27 (Invitrogen), Glutamax (Invitrogen), Heparin (2 *μ*g/ml) (Sigma-Aldrich Chemie GmbH, Taufkirchen, Germany) and the growth factors EGF (20 ng/ml; PromoCell, Heidelberg, Germany) and bFGF (20 ng/ml; Provitro, GmbH, Berlin, Germany) were used.

### Flow cytometry

For FACS analysis, all washing and staining steps were performed in phosphate-buffered saline containing 5% of fetal calf serum. Cells were incubated with the respective antibodies (see ‘Antibodies' section) for 1 h on ice in the dark. Appropriate single-stained and isotype controls were performed. All antibodies were used at the dilution recommended by the manufacturer or if not given otherwise 1 *μ*g per 100 *μ*l of sample volume. For exclusion of death cells, samples were stained with Hoechst 33342 (Invitrogen). For compensation, CompBeads (Beckton Dickinson, Heidelberg, Germany) were used according to the manufacturer's recommendations. Measurements were performed at a FACS Canto II (405 nm-488 nm-633 nm laser setup, Beckton Dickinson) using the FACS Diva software (Beckton Dickinson). At least 10 000 events were recorded. Results were analyzed using FlowJo (Tree Star, Ashland, Orlando, USA).

For FACS sorting, all washing and staining steps were performed in phosphate-buffered saline containing 2% B27 serum-free supplement (Gibco, Thermo Fisher Scientific GmbH, Schwerte, Germany) and 1% of human IgG (Gammunex, Talecris Biotherapeutics, Commons Research Triangle Park, NC, USA) under sterile conditions. Co-staining with anti-CD45-APC-Cy7 (eBioscience, Inc./Affymetrix, San Diego, CA, USA) (20 *μ*l/test) was performed to exclude myeloid cells. To exclude dead cells, the samples were stained with propidium iodide (BD Bioscience, Heidelberg, Germany). Sorting was performed on FACSAria II/III (BD Bioscience) at the Flow Cytometry Core Facility of the German Cancer Research Center (DKFZ).

### Tumorsphere assays, limiting-dilution assays (LDA) and treatment

Five hundred cells per well (or down to one cell per well for limiting dilution) were plated in complete neurobasal medium, and sphere formation was measured after 10 days. For LDAs, stem cell frequencies were calculated in R using the *limdil* function in the *statmod* package. In the treatment experiments, TMZ (10 *μ*M) and APG101 (10 *μ*g/ml) were added to the culture medium.

### CD95L-ELISA

GBM cells were treated with DMSO (Co) or with Temodal (TMZ, 100 μM) for 24 h, 4 days and 7 days. Supernatants and cell lysates were collected and analyzed by CD95L-ELISA (R&D, Cat. No. DFL00, Minneapolis, MN, USA) following the manufacturer's protocol.

### Scoring of immunostained paraffin sections

Consecutive sections of formalin-fixed paraffin-embedded primary GBMs and corresponding recidives from a total of 27 patients were stained against CD95 and CD95L. Stained sections were scored 1–3 for positive area (1: positive <1/3; 2: 1/3≤positive≤2/3; 3: positive >2/3) and intensity (1: weak; 2: medium; 3: strong). Combined scores for area and intensity of CD95 and CD95L were calculated by addition of the area and intensity scores. Relative scores for CD95 and CD95L were calculated by subtraction of the combined score of the recidive tumors from the combined score of the primary tumors.

### Proximity ligation assay

Duolink reagents (Sigma, Cat. Nos. DUO92101-1KT, DUO92009-1KT and DUO92010-1KT) were used following a slightly modified protocol. In brief, 1 × 10^6^ GBM cells (T6 CD95-WT and T6 CD95-mut) were treated with CD95L-T4 (40 ng/ml) for 10 min at 37 °C or left untreated as a control (Co). Cells were collected and fixed with 2% PFA for 10 min at RT. After washing with PBS, the cells were permeabilized with 0.5% Triton/PBS for 15 min at RT. Cells were incubated for 72 h with the respective primary antibodies (see ‘Antibodies' section) diluted in 0.5% Triton plus 2% B27 in PBS. After washing, the procedure was followed as described in the manufacturer's protocol.

### Immunoprecipitation

Cells (1 × 10^7^) were treated with 40 ng/ml CD95L-T4 for 5, 10 and 15 min (or left untreated as control) at 37 °C. After washing twice with PBS plus phosphatase inhibitors (NaF, NaN3, pNPP, NaPPi, *β*-Glycerolphosphate, 10 mM each and 1 mM orthovanadate), cells were lysed in IP Lysis Buffer (20 mM Tris/HCl, pH 7.5, 150 mM NaCl, 2 mM EDTA, 1 mM phenylmethylsulfonyl fluoride, protease inhibitor cocktail (Roche), 1% Triton X-100, 10% glycerol, and phosphatase inhibitors (NaF, NaN3, pNPP, NaPPi, ß-Glycerolphosphate, 10 mM each and 1 mM orthovanadate). Protein concentration was determined using a BCA Kit (Pierce, Thermo Fisher Scientific GmbH, Schwerte, Germany), and 500 *μ*g of protein was used as input. IP of CD95 or phospho-tyrosine was performed overnight with either an anti-CD95- (Apo-1, Apogenix, Heidelberg, Germany) or an anti-phospho-tyrosine biotinylated (Millipore/Merck Chemicals GmbH, Darmstadt, Germany) antibodies and 40 *μ*l protein-A Sepharose Beads and the corresponding isotype control.

Beads were washed five times with 20 volumes of lysis buffer, mixed with 40 *μ*l of 2 × Laemmli buffer and analyzed on 10% SDS-PAGE. Afterwards, proteins were transferred onto nitrocellulose membranes by semi-dry turbo-blotting. The non-specific binding sites on the nitrocellulose membrane were blocked by incubation with 5% skim-milk powder in PBS/Tween for 1 h. Later, the primary antibodies (anti-P85, Millipore; anti-SFK, Cell Signaling Technology Europe (B.V./New England Biolabs, ZA Leiden, The Netherlands); anti-CD95, Cell Signaling and anti-phospho-tyrosine, Millipore) diluted in 5% skim milk powder in PBS/Tween were incubated at 4 °C overnight. After washing again, the membranes were incubated for 1 h with the corresponding secondary antibody conjugated with horseradish peroxidase (HRP). The HRP signal was visualized by incubation with ECL solution and exposure to X-ray films (Hyperfilm, Amersham, Freiburg, Germany).

### RNA isolation

RNA isolation was performed using the mirVana Kit (Ambion, Thermo Fisher Scientific GmbH, Schwerte, Germany) following the manufacturer's total RNA purification protocol.

### EMT-qPCR array

RNA from sorted tumor samples was converted into cDNA using the SuperScript VILO cDNA Synthesis Kit (Life Technologies, Thermo Fisher Scientific GmbH, Schwerte, Germany) following the manufacturer's protocol.

Later, cDNA was mixed with RT^2^ SYBR Green qPCR Mastermix (SABioscienceGermany — QIAGEN GmbH, Hilden, Germany Cat. No. 330522) and loaded on either 96-well or 384-well RT^2^ Profiler Epithelial to Mesenchymal Transition PCR Array plates (Qiagen, GmbH, Hilden, Germany Cat. No. PAHS-090Z; PAHS-090ZE-4). The arrays were run as technical triplicates either on an ABI 7500 Real-Time PCR-System (Applied Bioscience/Thermo Fisher Scientific GmbH, Schwerte, Germany) for the 96-well plates or an CFX384 Real-Time PCR-System (Bio-Rad Laboratories GmbH, München, Germany) following the manufacturers' protocols, and the results for individual samples were averaged between the triplicates. Primary analysis was performed using an Excel sheet (Qiagen GmbH) provided by the manufacturer, and gene expression was normalized to ACTB expression.

Expectations regarding an upregulation or downregulation in EMT were annotated by the manufacturer's guidelines for some of the genes in the array and was complemented by information derived from searching Pubmed for non-annotated genes.

### Generation of heat maps

To present the EMT-qRT-PCR Array data from four different patient samples as well as GBM cells lentiviral induced with CD95-WT or a CD95 tyrosine-to-alanine mutant (CD95-mut), a heat map for each gene known to be upregulated during EMT was generated. The heat map represents the log_10_ of the ratio between the ACTB normalized expression of CD95^high^ and CD95^neg^ or CD95^WT^ and CD95^nut^ sorted cells from each tumor sample.

### Antibodies

The following antibodies were used for flow cytometric stainings: Anti-human CD95 (APO1, Apogenix), labeled with LightningLink APC-Cy7 (Innova Biosciences Ltd., Cambridge, UK); Mouse IgG Isotype Control (Sigma Aldrich), labeled with LightningLink APC-Cy7; Anti-human APG101 (Apogenix), labeled with LightningLink APC (Innova); Rabbit IgG XP Isotype Control (Cell Signaling), labeled with LightningLink APC (Innova); Anti-human CD44 PE-Cy7 (eBioscience); Rat IgG2b K Isotype Control PE-Cy7 (eBioscience); Anti-human CD15 PerCP (BioLegend); Mouse IgG1 K Isotype Control PerCP (BioLegend, San Diego, CA, USA); Anti-human CD133 PE (Miltenyi Biotec GmbH, Bergisch Gladbach, Germany); and Mouse IgG1 Isotype Control PE (Miltenyi).

The following antibodies were used for immunohistochemistry on paraffin-embedded tissue: Anti-human CD95 (Apogenix), anti-human CD95L (G24BD), biotinylated anti-mouse and anti-rabbit secondary antibodies, and anti-streptavidine-alkaline phosphatase.

The following antibodies were used for PLA: Anti-human CD95 (EnzoLife Sciences, Bergisch Gladbach, Germany; Enzo Life Sciences GmbH, Lörrach, Germany) and anti-phospho-Tyrosine biotinylated (Millipore).

The following antibodies were used for IP and immune blotting: anti-human CD95 (APO-1, Apogenix), labeled with LightningLink Biotin (Innova), anti-phospho-Tyrosine biotinylated (Millipore), anti-human P85 (Millipore), Sfk (New England Biolabs/Cell Signaling Technology), anti-human CD95 (Fas4C3, BioLegend), anti-mouse HRP, and anti-rabbit HRP (Dianova Gmb, Hamburg, Germany).

### General statistics

Sample names, group sizes and statistical tests are indicated in the respective figure/figure legends. *P*-values: *P≤0.05, **P≤0.01, ***P≤0.001. Data are presented as mean±S.E.M. Box whisker charts display minimum to maximum values (whiskers) and 25% and 75% percentiles (box). If not stated otherwise, data visualization and analysis was performed with GraphPad Prism 6 (GraphPad Software Inc., La Jolla, CA, USA).

## Abbreviations

APG101: CD95-Fc fusion protein
CD133: prominin-1
CD95-mut: CD95-tyrosine-to-alanine mutant
CD95-WT: CD95-Wildtype
CD95L: CD95 Ligand
CDKN2A: Cyclin dependent Kinase 2A
CNS: central nervous system
CSCs: cancer stem cells
DCX: doublecortin
OSX: osterix
EphA2: ephrin A2
EphA3: ephrin A3
FACS: flow activated cell sorting
GBM: glioblastoma multiforme
GSEA: gene set enrichment analysis
IP: immunoprecipitation
ITGA6: integrin α6
LDA: limiting dilution assay
MEF: myeloid elf-1 derived factor
ncEMT: non classical epithelial to mesenchymal transition
NF1: neurofibromatose 1
NSCs: neural stem cells
PLA: proximity ligation assay
qPCR: quantitative real time PCR
SFK: Src family kinase
SSEA-1: stage specific embryonic antigen 1
TCGA: the cancer genome atlas
TFGβ: transforming growth factor beta
TMZ: temozolomide
TNFRSF: TNF receptor super family
TNFα: tumor necrosis factor alpha
VIM: Vimentin
