# Supplementary material for: CD95 maintains stem cell-like and non-classical EMT programs in primary human glioblastoma cells
Source: Cell Death Dis. 2016 Apr 28;7(4):e2209–. doi: 10.1038/cddis.2016.102 (PMC4855647; doi:10.1038/cddis.2016.102)
Supplement: Supplementary Information [file cddis2016102x1.docx]

**SUPPLEMENTAL INVENTORY**

Supplemental information contains 3 figures, 3 tables and 2 movies.

**Figure S1** relates to Figure 1 and shows overall survival of TCGA dataset patients stratified by TNFR.

**Figure S2** relates to Figure 1 and shows survival of TCGA dataset patients stratified by CD95 expression within the respective GBM subtypes.

**Figure S3** relates to Figure 3 and provides supporting *in vivo* data.

**Figure S4** relates to Figure 6 and shows additional IP experiments from another patient sample and the correlation of the CD95 with EMT related genes on expression Data of the TCGA dataset.

**Table S1** relates to Figure 2 and lists all ranked genes from the GSEA analysis. The table is available as a separate .xls file.

**Table S2** relates to Figure 6 and provides additional clinical data for the patients from whom the slides analyzed in this figure have been derived. The table is available as a separate .xls file.

**Table S3** relates to Figure 7 and displays ΛCT values, fold change and p-values for all samples and genes in the RT^2^ EMT PCR Arrays. The table is available as a separate .xls file.

**Movies M1 and M2** are related to Figure 3 and show representative stacks of MRI pictures of CD95^high^ (M1) and CD95^neg^ (M2) DKFZ-GBM30 orthotopic xenograft tumors. Both files are uploaded separately as .mov files.

**Figure S1. Overall survival anaylsis for TNFRs according to the TCGA dataset.**

Kaplan-Meier curves showing overall survival in TCGA glioblastoma patients grouped according to the expression of the TNFR-family members. (TNFRSF1A, p=0.0255; TNFRFS1B, p=0.00537; TNFRFS11B, p=0.0173; TNFRFS12A, p=0.0437; TNFRFS14, p=0.0297, log-rank test). Low expression: below mean – 1 S.D.; Intermediate expression: mean +/- 1 S.D.; High expression: above mean + 1 S.D..

**Figure S2. Subtype-specific survival analysis for TCGA GBM patients.**

**A-D |** Kaplan-Meier curves for overall survival in TCGA glioblastoma patients grouped by subtype and CD95 expression (A: classical, p=0.557; B: neural, p=0.727; C: mesenchymal, p=0.973; D: Proneural **p=0.00112, log-rank test). Low CD95: below mean – 1 S.D.; Intermediate CD95: mean +/- 1 S.D.; High CD95: above mean + 1 S.D..

**Figure S3. Supporting *in vivo* data.**

**A |** Dot plot demonstrating sorting gates for DKFZ-GBM30 cells used for xenotransplantation in Figure 3. **B |** Representative pictures of H&E stained CD95^high^ and CD95^neg^ injected brains and corresponding MRI images illustrating the correlation between histological and MRI measurements (scale bars: 1mm). **C |** After two rounds of subcutaneous xenografting 1x10^3^ CD95^neg^ or CD95^high^ cells (DKFZ-GBM39) were injected into the striatum of SCID beige mice (n=8 for both groups). **D |** Tumor growth monitored by T2-weighted MRI. **E |** Tumor volumes at the end of the MRI period 19 weeks after injection. **F |** Kaplan-Meier curve showing the survival of the animals during the experiment.

**Figure S4. Additional CD95/P-Tyrosine immunoprecipitation and association between CD95 and known EMT marker genes in the TCGA dataset.**

**A |** Immunoprecipitation for CD95 and P-Tyrosine in GBM cells derived from another, independent patient sample and stimulated with CD95L-T4. Blots were probed with anti-P85 (regulatory PI3K-subunit), anti-Sfk, anti-CD95 or anti-P-Tyrosine antibodies respectively. **B-E |** Correlation of CD95 expression and known EMT markers in the TCGA GBM dataset. (n = 519 patients, R: Pearson’s correlation coefficient, p: probability for no or negative correlation).
