# Supplementary figures and images for: CD95 maintains stem cell-like and non-classical EMT programs in primary human glioblastoma cells
Source: Cell Death Dis. 2016 Apr 28;7(4):e2209–. doi: 10.1038/cddis.2016.102 (PMC4855647; doi:10.1038/cddis.2016.102)

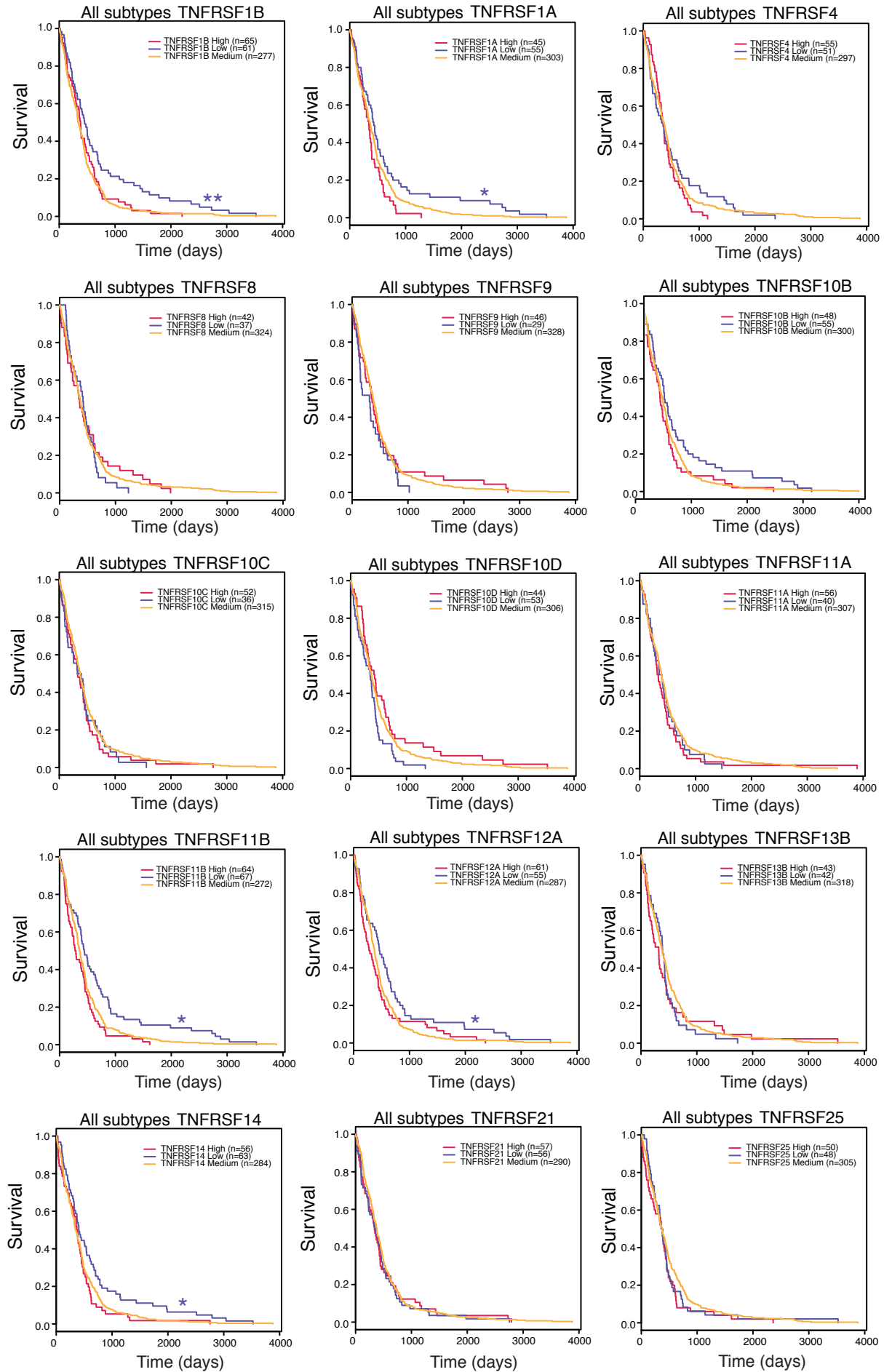

Supplement: Supplementary Figure 1 [file cddis2016102x2.pdf]

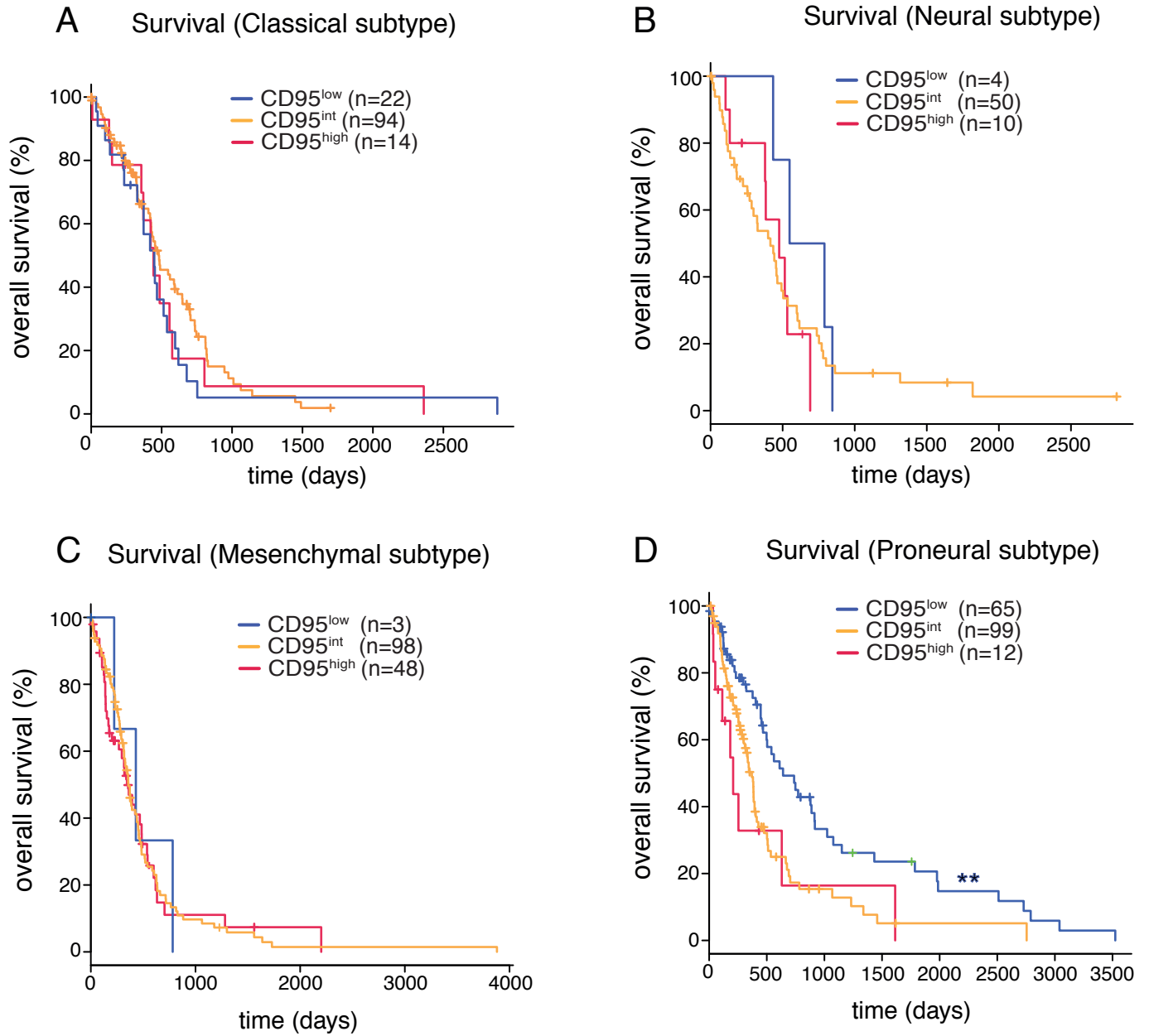

Supplement: Supplementary Figure 2 [file cddis2016102x3.pdf]

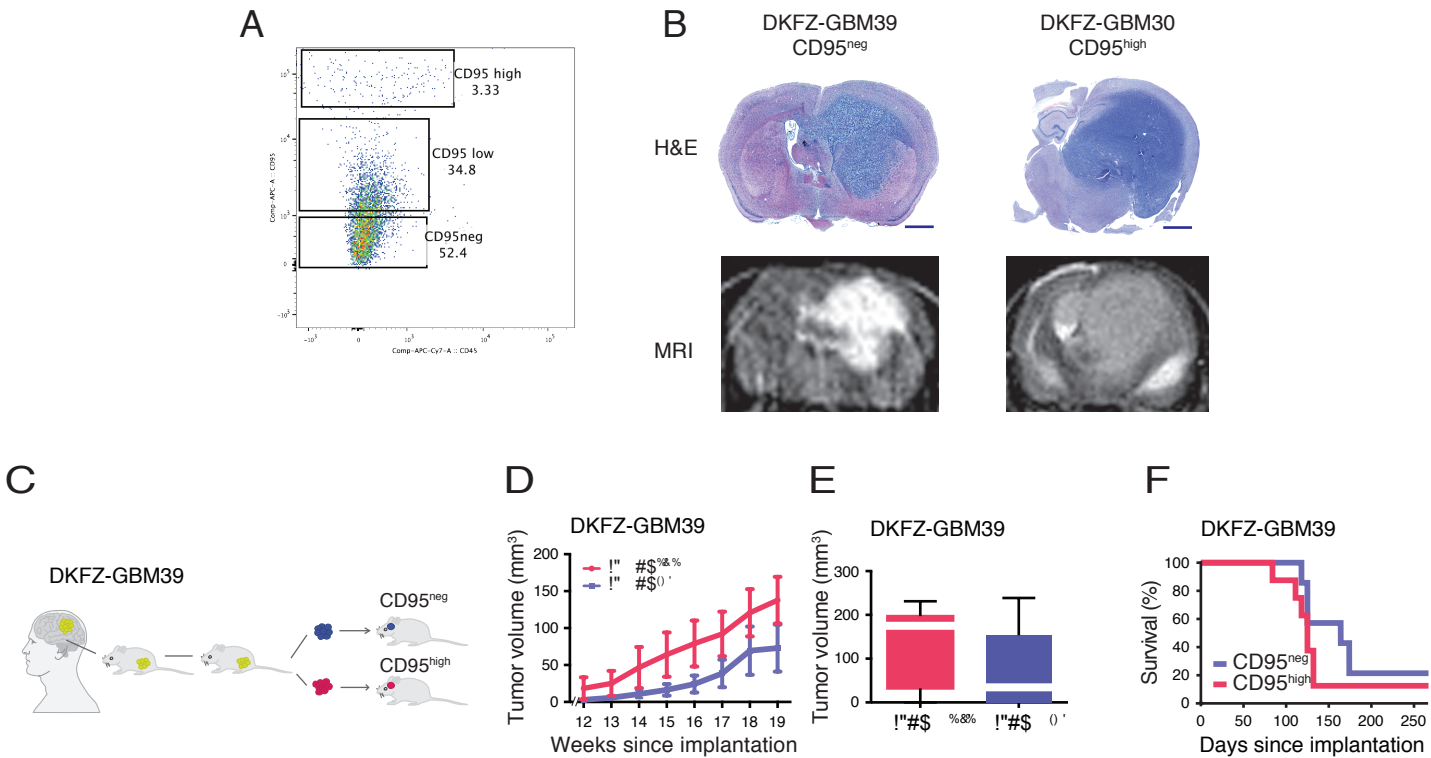

Supplement: Supplementary Figure 3 [file cddis2016102x4.pdf]

**A**

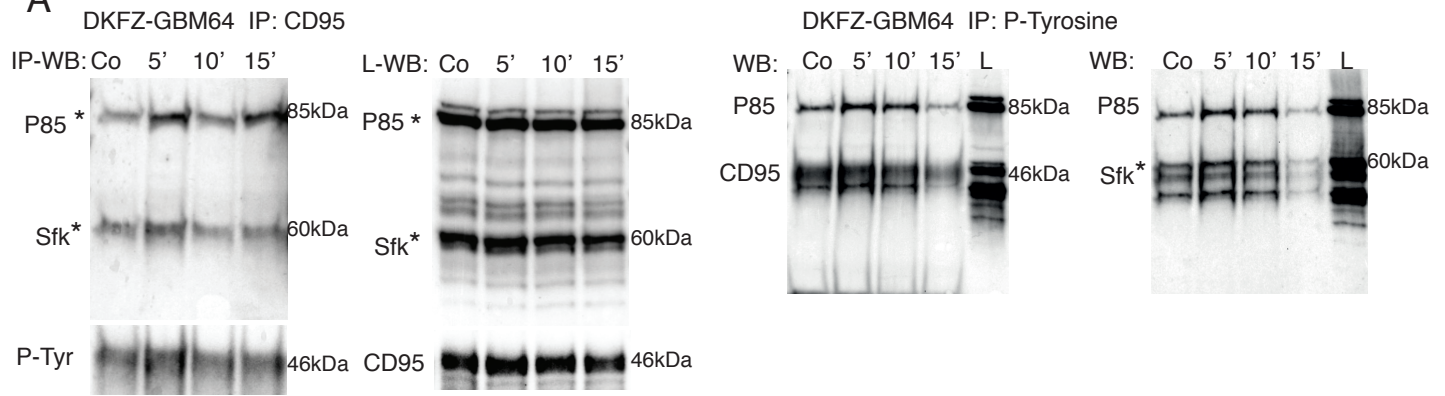

**B**

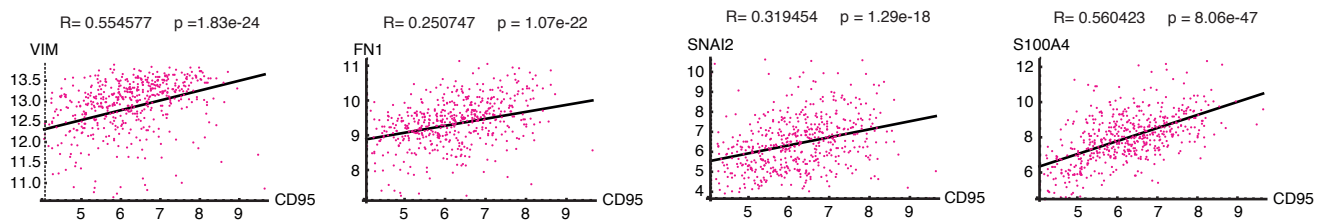

Supplement: Supplementary Figure 4 [file cddis2016102x5.pdf]
